# Pharmacogenomics of Novel Direct Oral Anticoagulants: Newly Identified Genes and Genetic Variants

**DOI:** 10.3390/jpm9010007

**Published:** 2019-01-17

**Authors:** Sri H. Kanuri, Rolf P. Kreutz

**Affiliations:** 1 Department of Clinical Pharmacology, Indiana University School of Medicine, Indianapolis, IN 46202, USA; srikanur@iu.edu; 2 Department of Medicine, Krannert Institute of Cardiology, Indiana University School of Medicine, 1800 N. Capitol Ave, MPC2, ME-400, Indianapolis, IN 46202, USA

**Keywords:** direct oral anticoagulant, dabigatran, rivaroxaban, apixaban, edoxaban, pharmacogenomics, genetic variants, SNPs, gene-drug interactions, genome guided therapy

## Abstract

Direct oral anticoagulants (DOAC) have shown an upward prescribing trend in recent years due to favorable pharmacokinetics and pharmacodynamics without requirement for routine coagulation monitoring. However, recent studies have documented inter-individual variability in plasma drug levels of DOACs. Pharmacogenomics of DOACs is a relatively new area of research. There is a need to understand the role of pharmacogenomics in the interpatient variability of the four most commonly prescribed DOACs, namely dabigatran, rivaroxaban, apixaban, and edoxaban. We performed an extensive search of recently published research articles including clinical trials and in-vitro studies in PubMed, particularly those focusing on genetic loci, single nucleotide polymorphisms (SNPs), and DNA polymorphisms, and their effect on inter-individual variation of DOACs. Additionally, we also focused on commonly associated drug-drug interactions of DOACs. *CES1* and *ABCB1* SNPs are the most common documented genetic variants that contribute to alteration in peak and trough levels of dabigatran with demonstrated clinical impact. *ABCB1* SNPs are implicated in alteration of plasma drug levels of rivaroxaban and apixaban. Studies conducted with factor Xa, *ABCB1*, *SLCOB1*, *CYP2C9*, and *VKORC1* genetic variants did not reveal any significant association with plasma drug levels of edoxaban. Pharmacokinetic drug-drug interactions of dabigatran are mainly mediated by p-glycoprotein. Strong inhibitors and inducers of CYP3A4 and p-glycoprotein should be avoided in patients treated with rivaroxaban, apixaban, and edoxaban. We conclude that some of the inter-individual variability of DOACs can be attributed to alteration of genetic variants of gene loci and drug-drug interactions. Future research should be focused on exploring new genetic variants, their effect, and molecular mechanisms that contribute to alteration of plasma levels of DOACs.

## 1. Introduction

Warfarin has been the main oral anticoagulant in clinical use since its discovery in 1954 by Wisconsin Alumini Research Foundation [1]. The underlying mechanism through which it exerts its anticoagulant effect are numerous, including inhibition of vitamin K epoxide reductase, attenuating vitamin K-dependent γ-carboxylation of coagulation factors II, VII, IX and, X.; and inhibition of protein C and S [2]. Despite the extensive clinical use of warfarin, it has many limitations such as slow onset of action, narrow therapeutic window, inadequate anticoagulation, increased risk of bleeding, frequent drug and food interactions, and need for frequent laboratory monitoring [3].

To offset these limitations, there was a need to develop alternative therapeutic options. New direct oral anticoagulants (DOAC) that are now used in clinical practice include dabigatran, rivaroxaban, apixaban, and edoxaban [4,5,6,7]. They are unique in acting on only a single coagulation factor (either thrombin or factor Xa) and target clot formation and fibrin deposition [3]. The current usage of DOACs has been increasing at a rapid rate over recent years [8]. Specifically, rivaroxaban usage increased from 0.13% to 13.87% from 2011 to 2014, whereas dabigatran usage increased from 1.3% to 7.6% from 2011 to 2014 [9].

The advantages of DOACs include high bioavailability, rapid onset of action, wide therapeutic window, no food interactions, few drug interactions, predictable pharmacokinetic and pharmacodynamic profiles, and no coagulation monitoring [2,3,10]. Due to the wide therapeutic range of DOACs, they are offered in fixed dosage schedules, which can be more convenient for patients [11].

The disadvantages of DOACs include higher cost, caution in liver and kidney impairment, a contraindication during pregnancy, and lack of clinical efficacy in thromboprophylaxis in setting of mechanical valves [2,10]. Currently approved uses of DOACs include prevention of stroke and thromboembolism in non-valvular atrial fibrillation, treatment of deep vein thrombosis (DVT) and pulmonary embolism (PE), and prevention of venous thromboembolism in knee/hip surgery [12]. Dabigatran may be preferred in patients with higher risk of stroke and thromboembolism and low risk of bleeding [13,14]. Apixaban may be preferred in elderly patients with high risk of thromboembolism, high bleeding risk, and moderate renal dysfunction [13,14]. Edoxaban may have advantages in patients with low thromboembolism and high bleeding risk [13]. Rivaroxaban once daily dosing regimen is the most convenient for indication of atrial fibrillation and may be preferred in patients with dyspepsia and medication compliance concerns [13,14].

Recently, specific antidotes have become available to treat major and life threatening bleeding related to DOACs, namely idarucizumab (mono-clonal antibody) for dabigatran and andexanet alpha (recombinant modified factor Xa) for factor Xa inhibitors [15,16]. Variation in therapeutic levels of warfarin can predispose the individuals to over-anticoagulation and increased risk of life threatening bleeding episodes [17,18]. The inter-individual variability in plasma levels of warfarin can be partly explained by single nucleotide polymorphisms (SNPs) of two genes encoding for CYP2C9 and VKORC1 [17,18,19,20]. Presence of genetic variants CYP2C9*2 and CYP2C9*3 reduce the clearance of *S*-warfarin can increase the risk of over-coagulation and bleeding episodes [17,18,19]. Furthermore, *VKORC1* genetic variants 1639 G>A and 1172 C>T increase warfarin sensitivity and are associated with increased risk of bleeding episodes [17,18,19].

Accordingly, knowledge of patient specific pharmacogenomics of warfarin can aid the clinician in appropriate dosing of warfarin in high risk cases. Previously conducted clinical trials revealed substantial clinical benefit with genotype guided warfarin therapy with lower out of range International Normalized Ratio (INR) values [21,22,23,24]. The Clinical Pharmacogenetics Implementation Consortium (CPIC) was created to help translate the results of pharmacogenomics testing into guidelines for appropriate dosage adjustments in patient specific clinical scenarios [24,25].

Pharmacogenomics of direct oral anticoagulants is currently a new area of research. Until now, very few genome wide association studies (GWAS) have been done to unravel the relevant genetic loci and genetic variants (SNPs) and their impact on drug metabolism and inter-individual variability of DOACs [26]. Current prescribing trends reveal that DOACs comprised 56.5% of oral anticoagulant prescriptions, with rivaroxaban most frequently prescribed, followed by apixaban and dabigatran, from 2012–2015 in the United Kingdom [27]. According to retrospective analysis of Medicare Part, D.; in 2015, DOACs claims comprised 31% of all anticoagulant claims, which showed a substantial increase of 127% in DOAC usage as compared to 2013 [28].

Although DOACs have predictable pharmacokinetics and pharmacodynamics and do not require routine coagulation monitoring per label, there have been recent reports of wider inter- individual variability in their plasma and drug responses [29,30]. Although several factors such as age, race, gender, smoking, and diet can lead to inter-individual variability of DOACs, presence of common genetic variants or drug-drug interactions may contribute to these differences [31].

This review article is to provide a brief overview of current approved indications, mechanism of action, pharmacokinetics, pharmacodynamic side effects, antidotes, drug-drug interactions, and drug gene interactions. The main purpose of this article is to focus on recently published pharmacogenomic studies conducted that have looked into the relationship between SNPs of common genetic variants and plasma drug levels of DOACs, namely dabigatran, rivaroxaban, apixaban, and edoxaban. Each drug review is followed by a table summary of recently published research articles and their impact on drug levels and clinical outcomes of all four DOACs. The discussed SNPs of genetic loci may provide information regarding personalization of therapy based on patient specific genetic variants for improving safety and efficacy of DOAC use in the general population.

## 2. Dabigatran

Dabigatran is a new direct oral anticoagulant agent, initially approved in Europe and later in 2010 by the Unites States Food and Drug Administration (FDA), for reducing the risk of stroke in non-valvular atrial fibrillation (AF) [32,33,34,35]. Later in 2014, the drug label was expanded to included prophylaxis against prevention of deep vein thrombosis and pulmonary thromboembolism in patients undergoing hip replacement [33,36,37]. Dabigatran is a reversible competitive inhibitor of thrombin that specifically inhibits both free and clot bound thrombin and thrombin induced platelet activation [32,38,39]. Dabigatran is administered as dabigatran etexilate orally, which is converted by esterases into the active form, dabigatran [32,39]. Major metabolites and metabolic pathways are demonstrated (Supplemental Figure S1). Remarkable pharmacokinetic features of dabigatran include rapid absorption, low bioavailability (3–7%), variable peak concentrations, attaining steady state concentration in 2–3 days, bi-exponential distribution phase, volume of distribution (50–70 L), 35% bound to plasma proteins, hepatic conjugation with glucuronide, and renal elimination (80%) [2,32,38,40,41,42].

Reilly et al. reported that dabigatran plasma concentration is mainly dependent on factors such as renal function, age, weight, and gender [43]. Additionally, the authors found that there was a fivefold variation in peak and trough levels for dabigatran doses 110 ng and 150 mg in the Randomized Evaluation of Long-Term Anticoagulation Therapy (RE-LY) trial conducted in 18,113 patients [43].

Since 80% of dabigatran is excreted by the kidneys, its half-life and dosing is dependent on kidney function and creatinine clearance [32,44]. The half-life of dabigatran ranges from 12–17 h and, it can be prolonged to 15–35 h in cases of renal insufficiency [32,44]. Although dabigatran can prolong prothrombin time (PT) and activated partial thromboplastin time (aPTT), these tests are not reliable for measuring dabigatran drug concentrations as they have poor correlation and poor sensitivity [45]. According to the International Council for Standardization in Hematology (ICSH) recommendations, liquid chromatography with tandem mass spectrometry (LC-MS/CS) is the gold standard test for measuring all DOAC concentrations [45].

Prolongation of aPTT correlates with low to moderate plasma concentration of dabigatran but it becomes less sensitive at supratherapeutic levels [46]. Thrombin Time (TT) is a very sensitive assay that can be used to assess the anticoagulant effect of dabigatran, although a normal TT time is indicative of minimal plasma levels of dabigatran [46,47]. In light of its favorable pharmacokinetic profile and predictable anticoagulant dose response, routine coagulation monitoring is not required per drug label in contrast to vitamin K antagonists [2,32,40,48].

Dabigatran usage was associated with low risk of dose dependent adverse effects such as dyspepsia, hepatic impairment, and bleeding events, supporting its safety profile [40,48].

Hemostatic agents such as activated recombinant factor VII (rFVIIa) and activated prothrombin complex concentrates (PCCa) may be used in the treatment of major bleeding episodes caused by supratherapeutic levels of dabigatran, although their efficacy is not well established in previously conducted clinical trials [32,38,40].

Recently, a monoclonal antibody, idarucizumab, was shown to reverse dabigatran induced anticoagulation by rapidly binding dabigatran and has been approved for dabigatran induced life threatening bleeding episodes [15]. Pharmacokinetic drug-drug interactions of dabigatran are mediated primarily by p-glycoprotein (p-gp), but not cytochrome P-450 enzymes [48,49]. Accordingly, caution should be exercised during the concomitant use of p-gp inducers such as rifampicin/rifampin (decreased concentration) and p-gp inhibitors such as ketoconazole, quinidine, amiodarone, and verapamil (increased concentration) with dabigatran [38,50]. Dabigatran and p-gp inhibitors should not be administrated together, particularly in severe renal impairment, as it can lead to high levels of dabigatran and life-threatening bleeding tendencies [38,50].

### 2.1. Pharmacogenomics

Dabigatran etelixate is an oral prodrug and is metabolized initially by intestinal carboxylesterase (CES2) enzyme to its intermediate metabolite dabigatran ethyl ester (M2) [51,52]. Later, M2 is converted to dabigatran (active form) by liver carboxylesterase (CES1) enzyme [51,52].

Bioavailability of dabigatran etelixate is influenced by the *ABCB1* gene, which encodes for p-glycoprotein (p-gp), an ATP dependent drug efflux transporter [29,30,53]. Even though routine coagulation monitoring is not required for use of dabigatran, substantial inter-individual variation in plasma concentration of dabigatran has been demonstrated. Several common genetic variants of *CES1* and *ABCB1* genetic loci have been identified in genome wide analyses, which may potentially account for some of the inter-individual variation in dabigatran plasma levels. Pare et al. conducted a genome wide pharmacogenomic analysis to characterize various *CES1* and *ABCB1* genetic variants associated with trough and peak levels of dabigatran [29].

### 2.2. CES1 Gene

Genetic variants of the liver carboxylesterase (*CES1*) gene, which catalyzes the conversion of M2 metabolite of dabigatran to active form, can regulate the pharmacokinetics of dabigatran.

One of the most important characterized is the *CES1* genetic variant SNP rs2244613 [29]. *CES1* SNP rs2244613 is associated with decreased dabigatran trough concentration and decreased risk of bleeding, a finding that may potentially impact efficacy of the drug during clinical use [29,53]. In general, high trough concentrations are more likely predictive of bleeding episodes as compared to high peak levels because lower trough levels can ensure intermittent strengthening of hemostatic plug formation at the site of vessel damage, thus resulting in less bleeding episodes [29]. None of the SNPs identified in this pharmacogenetic study demonstrated significant association with increased pro-thrombotic risk and no increase in incidence of ischemic events or systemic embolism [29]. *CES1* rs2244613 carriers were shown to have lower risk of bleeding episodes with dabigatran as compared with warfarin and non-carriers [53]. Additionally, minor allele carriers of *CES1* rs2244613 were also shown to have less bleeding episodes with lower doses (110 mg twice daily) as compared to higher doses (150 mg twice daily) [53]. Patients who are heterozygous for SNP rs2244613 have been shown to have 15% lower risk of bleeding, whereas patients who are homozygous for SNP rs2244613 demonstrated 28% lower incidence of bleeding with dabigatran treatment as compared to warfarin [54]. There were no reports of increased adverse thrombotic episodes in patients with *CES1* SNP rs2244613, who had lower bleeding risk among subjects studied in the RE-LY trial [29]. It has been reported that lower trough levels of dabigatran leads to formation of more stable hemostatic formation that reduces the risk of bleeding tendencies with *CES1* SNP rs2244613 [29].

In patients receiving dabigatran prophylaxis after orthopedic surgery, higher trough levels were associated with CC genotype of *CES1* rs2244613 (34.6 (32.2–36.9) ng/mL), whereas lowest trough levels were associated with AA genotype of *CES1* rs2244613 (17.1 (12.0–25.4) ng/mL), although these findings were not statistically significant [52].

Since *CES1* genetic locus has more than 2000 genetic variants, other mutants were also characterized to assess their effect on dabigatran peak and trough concentrations [51,54].

Another *CES1* SNP (rs8192935) has been associated with variability of both peak and trough concentrations of dabigatran with no clinical impact on bleeding risk [29,53]. The presence of *CES1* SNP rs819295 was associated with a 12% decrease in peak plasma levels of dabigatran [54]. It has been suggested that linkage disequilibrium of *CES1* rs8192935 with unknown allelic variants may contribute to alterations in plasma levels and may regulate dabigatran anticoagulant efficacy [30]. In patients with atrial fibrillation receiving dabigatran for anticoagulant prophylaxis, CC genotype of *CES1* SNP rs8192935 was associated with higher plasma levels of dabigatran (86.3 ng/mL), as compared to T genotype (62.1 ng/mL) [30,52].

*CES1* SNP rs71647871 (or G14E) loss of function variant attenuates the metabolism of dabigatran and its metabolites (M1 and M2) [51]. Specifically, mean activation rates of dabigatran, M1, and M2 in the carriers of the *CES1* G14E mutant were lower at 53%, 43%, and 37%, respectively, as compared to non-carriers (100%) [51]. *CES1* variants rs2244613 and rs8192935 were not associated with dabigatran levels in this study [51]. Of note, metabolism of dabigatran and its metabolites were significantly higher in female livers as compared to male livers [51].

### 2.3. ABCB1

One of the most important *ABCB1* genetic variants is rs1045642, which was found to be significantly associated with peak dabigatran concentration and increased incidence of bleeding episodes [52]. Particularly, patients with TT genotype of rs1045642 experienced higher peak dabigatran levels and higher risk of bleeding (relative risk: 1.72; 95% confidence interval: 0.92–3.22) than patients with CC genotype of rs1045642, although this is not a significant finding [52]. The peak plasma concentration was higher in subjects with TT genotype (291.8 (193.6–345.0) ng/mL) than in subjects with CC genotype (124.1 (79.9–177.7) ng/mL) (*p* = 0.008), but there was no significant difference in trough levels [52]. Another well studied *ABCB1* variant (rs4148738) has been associated with up to 12% increase in peak concentration of dabigatran without significant impact on bleeding risk [29,53]. In patients receiving dabigatran for venous thromboembolism prophylaxis after total knee replacement surgery, equilibrium peak levels were 218.7 (143.4–310.7) ng/mL for GG genotype and 130.7 (102.2–249.1) ng/mL for GA genotype with respect to ABCB1 rs4148738 polymorphism [52]. Third, *ABCB1* variants rs2032582 (C.2677G>A/T), rs1045642 (c.3435C>T), and rs1128503 (1235C>T) were also studied. These three SNPs occur as haplotype together and they can be related to each other by linkage disequilibrium [55]. There appears to be limited impact of homozygous, heterozygous, and wild type variants *ABCB1* haplotype 1235–2677–3455 on pharmacokinetics of dabigatran [55]. A summary of pharmacogenetic studies performed with dabigatran is provided in Table 1.

## 3. Rivaroxaban

Rivaroxaban is approved for use in non-valvular atrial fibrillation, treatment of deep vein thrombosis, pulmonary embolism, cardiovascular disease, as well as prevention of thromboembolism after orthopedic surgery [56,57]. The principal mechanism of action of rivaroxaban is through inhibition of factor Xa, resulting in a blockade of intrinsic and extrinsic coagulation pathways [58,59,60]. Rivaroxaban is a small molecule oxazolidinone that specifically binds to S1 and S4 pockets of factor Xa [58]. It has 10,000 times more selectivity than any other related serine proteases and binds to free and clot bound factor Xa [58]. Some of the important pharmacokinetic properties of rivaroxaban include rapid absorption, 100% bioavailability, 90% protein bound, 1.46 L volume of distribution, 5–9 h half-life, and elimination through renal and fecal routes [57,59,60,61]. Even though bioavailability of 100% has been reported by some previous authors, it varies according to drug dosage and food administration. The bioavailability of rivaroxaban depends on the dose with 10 mg achieving 80–100% bioavailability whereas 20 mg dose leads to 66% bioavailability [61]. Co-administration of food with 15–20 mg rivaroxaban dose resulted in higher bioavailability and substantial increase in area under curve (AUC) and maximal plasma concentration (Cmax) [61,62].

Rivaroxaban is the main active form, with onset of action and peak plasma concentration occurring within 1–4 h [57,58,59,62,63]. Major metabolites and metabolic pathways are demonstrated in Supplemental Figure S2. Maximum anticoagulant efficacy is achieved within 1–4 h and its antiXa activity returns to baseline in 24–48 h [63]. Rivaroxaban dosing at 5–80 mg can result in 20–80% inhibition of factor Xa activity [57,58,59].

Area under curve (AUC) and maximal plasma concentration of rivaroxaban are substantially increased in renal impairment, hepatic impairment, and in patients older than 75 years [57,58,59,62,63]. Major bleeding episodes caused by supratherapeutic levels of rivaroxaban may be treated with discontinuation of therapy, PCCa, and andexanet alpha [54,64,65]. Andexanet alpha is FDA approved for treatment of life threating and uncontrolled bleeding episodes with rivaroxaban, but it is associated with adverse effects such as deep vein thrombosis, pulmonary embolism, myocardial infarction, and ischemic stroke [16].

Drug-drug interactions of rivaroxaban are mediated by CYP450 enzymes and p-gp [56,57,60]. Particularly, strong inhibitors of CYP450 enzymes and p-gp should be avoided in combination with rivaroxaban as they may increase the plasma concentration and may lead to increased risk of bleeding tendencies [56,57,60,61]. Moderate inhibitors of CYP450 enzymes and p-gp can be administrated in combination with rivaroxaban.

Particularly in high risk patients such as those with renal failure, bleeding complications, stroke and prior to major surgery, accurate measurement of peak and trough levels can be performed through high performance liquid chromatography-mass spectrography (HPLC-MS) which is only available in a few laboratories [66]. According to International Council for Standardization in Hematology (ICSH) recommendations, laboratory assessment of rivaroxaban can be accurately performed with LC/MS-MS (liquid chromatography with tandem mass spectrometry) and drug calibrated anti-FXa, but not PT and aPTT as they are not reliable for measuring therapeutic drug concentrations [45].

### 3.1. Pharmacogenomics

As previously described, rivaroxaban is metabolized by CYP450 isoenzymes (CYP3A4, CYP2C8 and CYP2J2), p-gp, and the efflux transporter breast cancer resistance protein (BRCP) [24,67,68]. Strong inducers of CYP3A4 such as rifampicin/rifampin, carbamazepine, phenobarbital, and phenytoin should not be co-administered with rivaroxaban as they result in a decrease in AUC and attenuated pharmacodynamics effects [60,61,69]. Conversely, co-administration of rivaroxaban with strong CYP3A4 and p-gp inhibitors such as ketoconazole, itracomazole, voriconazole, posaconazole and ritonavir can result in an increase in AUC, Cmax, and increased risk of bleeding tendencies [60,61,69]. Moderate CYP3A4 and p-gp inhibitors such as erythromycin and clarithromycin can be co-administered with rivaroxaban as their clinical impact is not clearly defined.

### 3.2. ABCB1

*ABCB1* gene polymorphisms, which encode for p-gp, were investigated in only a few clinical studies to assess whether they account for differences in pharmacokinetics of rivaroxaban [55]. P-glycoprotein and BRCP are mainly responsible for active renal secretion of rivaroxaban [67]. There are more than 100 polymorphisms of *ABCB1*, among which rs2032582 (C.2677G>T) and rs1045642 (C.3435C>T) have been shown to affect rivaroxaban metabolism [54,55,67]. SNPs (C.2677G>T) and (C.3435C>T) exhibit linkage disequilibrium and are frequently documented to exist as haplotypes. The frequency of TT haplotype in the Caucasian population is around 25–40% [67]. According to a recently published case report, patients who are homozygous to haplotype (C.2677G>T; TT and C.3435C>T; TT) may have higher plasma levels, Cmax, half-life, and increased risk of bleeding complications [67]. Pharmacogenomic screening of this haplotype mutation may be warranted particularly in patients with risk factors such as renal impairment and *CYP3A5* inhibitors [67]. Another *ABCB1* genetic variant (1236 C>T) was also studied, and the combined *ABCB1* haplotype (1236-2677-3435) did not have any significant effect on pharmacokinetic metabolism of rivaroxaban [55]. A summary of pharmacogenetic studies performed with rivaroxaban is listed in Table 2.

### 3.3. CYP3A

Sychev et al. reported a significant correlation between *CYP3A* activity and rivaroxaban peak and trough levels in patients with deep vein thrombosis [70]. However, no significant correlation between *CYP3A* activity and treatment parameters in deep vein thrombosis patients treated with rivaroxaban were found [70]. A summary of genetic variants analyzed for rivaroxaban is provided in Table 2.

## 4. Apixaban

Apixaban is widely used in prevention of stroke in non-valvular atrial fibrillation and treatment of deep vein thrombosis and pulmonary embolism [71].

Moreover, apixaban is considered a cost effective therapeutic option in comparison to warfarin across different economic evaluations, such as willingness to pay thresholds, discount rates, medical costs, and healthcare systems for stroke prevention in non-valvular atrial fibrillation [72]. It has also been shown to be equally efficacious as compared to enoxaparin and warfarin in prevention of recurrent venous thromboembolism in adults with acute VTE [73,74]. It is used for treatment of deep vein thrombosis and pulmonary embolism, as well as prevention of venous thromboembolism after orthopedic surgery [73]. Its main mechanism of action is through reversible inhibition of factor Xa. It specifically binds and inhibits both free and bound factor Xa which ultimately results in reduction in the levels of thrombin formation [75].

Some of the important pharmacokinetic features of apixaban include 50% bioavailability, peak levels reached within 1–2 h, 87% bound to plasma proteins, 21 L volume of distribution, a half-life of 12 h, hepatic *CYP450* metabolism, and 25% renal excretion [73,75,76]. Apixaban is mainly metabolized by *CYP3A4* with minor contributions from *CYP1A2, CYP2C8, CYP2C9, CYP2C19 and CYP2J2* isoenzymes [54]. Some of the major metabolic pathways of apixaban include o-demethylation, hydroxylation, and sulfation, with o-demethyl apixaban sulphate being the major metabolite (Supplemental Figure S3) [77]. The principal modes of elimination of apixaban include fecal (56%) and renal pathways (24.5%) [77]. In view of multiple modes of metabolism and elimination, it appears to be safe to use in patients with renal or hepatic impairment [77].

Major bleeding episodes with apixaban may be treated with prothrombin complex concentrates (PCCa) and andexanet alpha [54,78].

### 4.1. Pharmacogenomics

Apixaban is a substrate for p-gp and *CYP450* enzymes (3A4) and thus caution should be exercised during concomitant administration of strong inducers and inhibitors of both metabolic pathways [50,75]. Strong inducers of CYP3A4 and p-gp such as rifampicin/rifampin, carbamazepine, phenytoin, and phenobarbital can reduce the plasma levels of apixaban and result in a decrease in its therapeutic effect. Conversely, strong inhibitors of CYP3A4 and p-gp such as ketoconazole, itraconazole, ritonavir, and clarithromycin can lead to supra-therapeutic levels of apixaban and excessive bleeding tendencies [50,75]. Moderate CYP3A4 and p-gp inhibitors such as erythromycin and clarithromycin can be co-administered with apixaban as their clinical impact is not clearly defined.

### 4.2. SULT1

A potentially important pharmacogenomic metabolic pathway is through sulfotransferases (SULTs) *SULT1A1 and SULT1A2*, which are responsible for sulfation of o-demethyl-apixaban to o-demethyl-apixaban sulphate [31,79,80]. *SULT1A1* is more potent than *SULT1A2* in sulfation of o-demethyl-apixaban [79]. O-demethyl-apixaban is the most prominent metabolite and represents 25% of estimated active apixaban [77].

Is it important to know that o-demethyl-apixaban sulphate does not possess any inhibitory activity against factor Xa that can contribute to anticoagulant efficacy of apixaban [79]. Three important allelic variants, SULT1A1*1 (wild type), SULT1A1*2, and SULT1A1*3 have been described [80]. Vmax of all the three allelic variants of SULT1A (SULT1A*1 > SULT1A*3 > SULT1A*2) varies and it accounts for differences in sulfation of active apixaban [81]. SULT1A*3 has moderate potential to affect anticoagulant effect of apixaban, whereas SULT1A*2 has very low potential effect on metabolism of apixaban (Table 3) [81].

These different alloenzymes have different enzymatic efficacies and may lead to different metabolite concentrations and variations in anticoagulant efficacy of apixaban [81,82]. The impact of common genetic variants of *SULT1A1* on apixaban metabolism in patients however has not been formally tested as of yet.

### 4.3. ABCB1

*ABCB1* rs4148738 is significantly associated with variability of peak levels of apixaban as compared to trough levels [83]. Particularly, AA genotype of rs4148738 had higher peak levels of apixaban as compared to carriers of variant G allele [83]. Specifically, peak levels of apixaban in carriers of *ABCB1* rs4148738 G variant are decreased by 26% in heterozygytes and 32% in homozygotes [83]. The authors concluded that p-glycoprotein may account for some of the genetic variation in peak plasma levels of apixaban [83]. A study by Valarevich et al. showed no significant association of *ABCB1* SNPs (rs1045642 and rs4148738), as well as CYP3A5*3 (rs776746) with pharmacokinetics of apixaban in patients with atrial fibrillation and stroke [84]. A summary of pharmacogenetic studies performed with apixaban is provided in Table 4.

## 5. Edoxaban

Edoxaban is a direct oral anticoagulant approved for prevention of stroke and systemic embolism in patients with non-valvular atrial fibrillation [2]. It has also shown efficacy in prevention of recurrent venous thromboembolism in patients undergoing total knee replacement surgery as compared to warfarin and enoxaparin [2,85]. Its mechanism of action is through competitive inhibition of factor Xa in a highly selective manner [3,86,87]. Inhibition of factor Xa activity is the main mechanism of action that leads to reduction in thrombin generation and thrombus formation, which also indirectly results in decrease in platelet activation [3,87]. It has been shown to prolong prothrombin time (PT), and activated partial thromboplastin time (aPTT), and to decrease thrombin generation in thrombin generation (TG) assay [3]. In animal models of venous stasis, edoxaban has been shown to inhibit factor Xa activity and thrombus formation [3,88]. In animal models of arterial thrombosis, edoxaban in combination with aspirin and clopidogrel has afforded additive antithrombotic effects with excessive bleeding tendencies only in combination with clopidogrel [3,89,90].

Some of the important pharmacokinetic parameters with edoxaban include rapid absorption through upper gastrointestinal tract (1–3 h), 62% bioavailability, peak plasma concentration reached within 1–2 h, 107 L volume of distribution, 40–59% plasma protein binding, half-life 10–14 h, total clearance 22 L/h with elimination by kidney, and hepatic metabolism [3,86,87,91]. Systemic exposure levels of edoxaban do not seem to be affected by food or hepatic impairment, but dosage adjustment is needed for renal impairment [87,91,92].

Edoxaban undergoes extensive metabolism through CES1, CYP3A4, and glucuronidation [91]. Edoxaban can be metabolized into various metabolites such as M1 (Hydrolysis), M3 (conjugation), M4 (CES1), M5 (CYP3A4), M6 (CYP3A4), and M7 (CYP3A4) (Supplemental Figure S4) [91]. All the metabolites, including the most abundant metabolite M4, do not significantly contribute to anticoagulant activity [91]. Metabolite M4 is a substrate for organic anion transporter 1B1 (OATP1B1). Changes in the levels of OATP1B1 affect edoxaban and M4 plasma concentration [93]. Bleeding caused by supra-therapeutic levels of edoxaban can be treated with factor VIIa concentrates, prothrombin complex concentrates, anti-inhibitor coagulation complex, andexanet alpha, and synthetic small molecule (PER977) [3,54].

### 5.1. Pharmacogenomics

Endoxaban is a substrate of p-gp and CYP3A4. Major drug interactions of edoxaban can be observed with strong and moderate inducers or inhibitors of both p-gp and CYP3A4 [3,26,86,87,91]. Co-administration with inhibitors of p-gp and CYP3A4 (ketoconazole, quinidine, verapamil, erythromycin, cyclosporine, and amiodarone) results in increase in peak levels and total exposure of edoxaban [3,86,87,91]. Administration of rifampicin/rifampin (strong p-gp inducer) along with edoxaban results in decrease in total exposure and peak levels [91]. Drugs such as aspirin, naproxen, digoxin, atorvastatin, and esomeprazole did not alter the peak levels, Cmax, AUC, and total exposure levels of edoxaban [3,91].

### 5.2. ABCB1

In a study recently conducted by Vandell et al., ABCB1* SNP (rs1045642; C3435T) and *SLCO1B1* SNP (rs4149056; T521C) were found to have no significant effect on the pharmacokinetics of edoxaban, although the same study showed a significant increase in plasma levels of edoxaban and slight elevation of M4 metabolite (*SLCOB1B1* C-allele carriers) with p-gp and OATP1 inhibitors [93].

### 5.3. CYP2C9 and VKORC1

In a recent randomized double blinded study comparing edoxaban with warfarin in the treatment of venous thromboembolism (Hokusai VTE), *CYP2C9* SNPs (rs1799853 and rs1057910) and *VKORC1* SNP (rs9923231) did not have any significant impact on the risk of major bleeding episodes or clinical response in subjects randomized to edoxaban [94]. In the randomized double blinded study comparing edoxaban with warfarin in patients with atrial fibrillation (ENGAGE AF-TIMI48), there was a trend towards the consistent long-term safety benefit of edoxaban over warfarin across genotypes [95]. Mega et al. also found that patients with *CYP2C9* (rs1799853 and rs1057910) and *VKORC1* (rs9923231) genotypes derived greater safety benefit from edoxaban compared with warfarin by identifying patients who are more likely to experience early bleeding with warfarin [95]. A summary of pharmacogenetic studies performed with edoxaban is provided in Table 5.

## 6. Conclusions and Future Perspective

Despite broad therapeutic windows of novel oral anticoagulants, there has been recognition that both pharmacogenomics as well as relevant drug-drug interactions can lead to variability in plasma drug levels [53]. In the case of dabigatran, *CES1* and *ABCB1* genes and their SNPs have been associated with variability in plasma drug levels of dabigatran, whereas *ABCB1* and *CYP3A4* is implicated in altering plasma drug levels of rivaroxaban. *SULTA1A*, a relatively novel candidate gene identified as important in the metabolism of apixaban, as well as known variants in *ABCB1* gene have been implicated in altering plasma drug levels of apixaban. While associations have been described between alterations in drug levels of most DOAC and important pharmacogenetic pathways, there has been fewer data showing linkage of variants with either bleeding or thromboembolic events. This review summarizes the clinical studies conducted for each drug. The lack of solid clinical outcomes data from large enough clinical trial cohorts limits the scope of recommendations drawn from the pharmacogenetic and pharmacokinetic studies.

For warfarin, the Clinical Pharmacogenetics Implementation Consortium (CPIC) was created for designing genotype-based dosing algorithms for appropriate dosage adjustments in patient specific clinical scenarios [24,25]. Before similar genotype-based dosing algorithms for DOACs could be generated, further prospective clinical studies would be needed.

**Figure 1:**
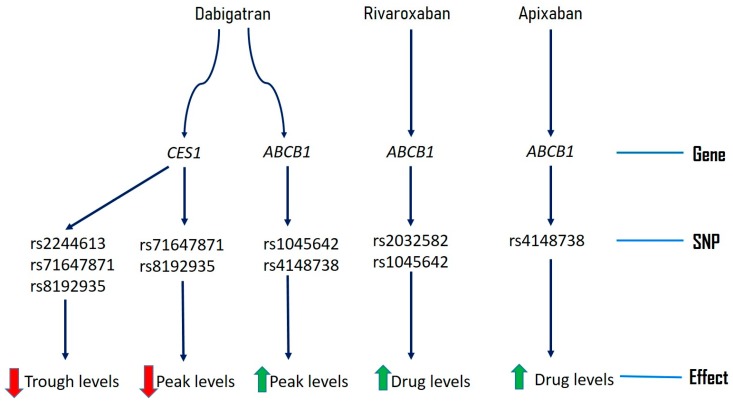
Pharmacogenomics of novel direct oral anticoagulants.

Genetic variants of genes including *ABCB1* and *CES1* might be responsible for some of inter-individual variability of DOACs. We have summarized genetic variants reported to be associated with altered drug levels of DOACs in Figure 1 and frequency in populations in Table 6. Sometimes these genetic variants might be in linkage disequilibrium with other unknown genetic variants, which needs to be taken into consideration for interpreting dug variability and anticoagulant efficacy of DOACs [30].

Apart from these variants, other factors such as rare mutations, epigenetic mechanisms, DNA methylation, and environmental factors might also contribute to inter-individual variation of DOACs [26]. Genome wide association studies are warranted for identifying additional genetic variants that are involved in metabolism and transport of DOACs and their impact on anticoagulant efficacy and adverse effects [26]. Since there are around 2000 genetic polymorphisms in the *CES1 gene*, a focused CES1 pharmacogenetic study may potentially uncover genomic variants that might influence the metabolism and clinical outcome of dabigatran usage [51]. Although *ABCB1* gene variants did not significantly influence the inter-individual variability of rivaroxaban plasma concentrations in some studies, future research is warranted to understand its role in combination with *CYP3A4* genetic polymorphisms [30,55,96]. *ABCG2* (BRCP efflux transporter), *CYP3A4/5*, and *CYP2J2* genetic polymorphisms should also be studied to understand their impact on inter-individual variability of rivaroxaban pharmacokinetics [54,67,96]. Genetic variants of sulfotransferase (*SULT1A1*), which is involved in metabolism of apixaban, needs additional clinical studies to ascertain its effect on clinical efficacy and side effects [79].

Direct oral anticoagulants demonstrate a wide therapeutic index and, while safer than Vitamin K antagonists, their use is associated with both adverse bleeding events as well as sub-therapeutic inhibition of coagulation.

In conclusion, pharmacogenomics of novel direct oral anticoagulants is a relatively new field of research. Important genetic variants affecting DOAC pharmacokinetics described in this review need further consideration. Further clinical studies are required to better understand the impact of variations in DOAC drug levels related to pharmacogenomics on actual clinical outcomes, both in terms of risk of bleeding as well as risk of diminished clinical efficacy (lack of protection from thrombotic events). Understanding the importance of genetic variants in metabolism and transport of novel DOACs might lead to effective personalized therapy and better clinical outcomes.

## 7. Executive Summary

### 7.1. Direct Oral Anticoagulants

- To offset the significant challenges posed by vitamin K antagonists (warfarin) such as narrow therapeutic index, drug interactions, and frequent coagulation monitoring newer direct oral anticoagulants (DOACs) were introduced.
- Four DOACs that are currently in clinical use include dabigatran, rivaroxaban, apixaban, and edoxaban.
- DOACs act on specific coagulation factors inhibiting thrombin generation.
- Currently approved indications of DOACs include prophylaxis against thromboembolism in non-valvular atrial fibrillation, treatment and prevention of deep vein thrombosis and pulmonary embolism, and secondary prevention in chronic coronary artery and peripheral vascular disease.
- DOACs are cost effective and improve quality of life as compared to warfarin in approved clinical conditions.

### 7.2. Dabigatran

- Dabigatran acts through reversible competitive inhibition of thrombin.
- It is administered as prodrug that is converted into active form dabigatran etelixilate by esterases.
- Principal drug interactions are mediated by p-glycoprotein.
- *CES1* and *ABCB1* gene loci and their SNPs are implicated in altering plasma peak and trough levels of dabigatran.

### 7.3. Rivaroxaban

- Rivaroxaban acts through inhibition of factor Xa.
- Principal drug interactions are mediated by CYP3A4 and p-glycoprotein.
- *ABCB1* and *CYP3A4* gene loci and their SNPs are implicated in altering plasma drug levels of rivaroxaban.

### 7.4. Apixaban

- Apixaban acts through reversible inhibition of factor Xa.
- Principal drug interactions are mediated by CYP3A4 and p-glycoprotein.
- *ABCB1* gene locus and its SNPs are implicated in altering plasma drug levels of apixaban.
- Sulfotransferases polymorphisms may potentially contribute to variability of apixaban metabolism.

### 7.5. Edoxaban

- Edoxaban acts through inhibition of factor Xa.
- Principal drug interactions are mediated by CYP3A4 and p-glycoprotein.
- Clinical trials failed to show significant association between *ABCB1, CYP2C9*, and *VKORC1* and plasma drug levels of edoxaban.

## 8. Conclusions

- Drug-gene interactions of DOACs are mainly mediated by genes *CES1*, *ABCB1*, and *CYP3A4* and their respective SNPs.
- Drug-drug interactions of DOACs are mainly mediated by CYP3A4 and p-glycoprotein.
  Further research including larger randomized clinical trials should be conducted to uncover other genetic variants and understand their impact on plasma drug levels and clinical efficacy of DOACs.

## Figures and Tables

**Table 1:** Common genetic variants associated with pharmacodynamics and pharmacokinetics of dabigatran.

**Peak Levels**	**SNP**	**Locus**	**Function**	***p*-Value**	**Change (Concentration)**	**Clinical Outcome**	**Ref.**	**Year**
	rs8192935	*CES1*	Intron	3.2 × 10^−8^	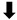	No significant association with clinical events	[29]	2013
	rs4148738	*ABCB1*	Intron	8.2 × 10^−8^	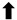	No significant association with clinical events	[29]	2013
	rs1045642	*ABCB1*	Intron	0.008	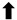	No significant difference	[52]	2018
	rs71647871 (G143E)	*CES1*	Intron	0.018	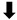	Not tested	[51]	2016
	2677–3455	*ABCB1*	Intron	0.58	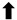	Not tested	[55]	2016
**Trough Levels**	**SNP**	**Locus**	**Function**	***p*-Value**	**Change**	**Clinical Outcome**	**Ref.**	**Year**
	rs2244613	*CES1*	Intron	1.2 × 10^−8^	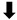	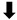 Bleeding	[29]	2013
	G14E	*CES1*	Intron	0.018	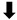	Not tested	[51]	2016
	rs8192935	*CES1*	Intron	0.023	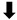	Not tested	[30]	2016
	rs2244613	*CES1*	Intron	0.04	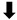	Not tested	[30]	2016

Peak and trough levels of Dabigatran and clinical outcomes associated with gene loci and their single nucleotide polymorphisms (SNPs) in recently conducted clinical studies.

**Table 2:** Common genetic variants associated with pharmacodynamics and pharmacokinetics of rivaroxaban.

Gene	Exon	SNP	DNA Polymorphism	Changes in Peak Plasma Levels	Study Population	Reference	Year
*ABCB1*	21	rs2032582	C.2677G>T	Increased	Case report	[67]	2016
*ABCB1*	21 26	rs2032582 rs1045642 Combined haplotype (2677–3435)	C.2677G>T C.3435C>T	Non-significant increase	Healthy volunteers	[55]	2016
*ABCB1*	26	rs1045642	C.3435C>T		Case report	[67]	2016
*ABCB1*	---	rs1128503 Combined haplotype (1236–2677–3455)	C.1236C>T	No Change	Healthy volunteers	[55]	2016

Peak levels of Rivaroxaban associated with gene loci and their single nucleotide polymorphisms in recently conducted clinical studies.

**Table 3:** Allelic variants of sulfomethyl transferase *SULF1A1* that affect the pharmacokinetic metabolism of apixaban.

SULT1A	Allelic Variants	Substitution	Whites	Blacks	Chinese	Reference
	SULT1A1*1	Wild type	65.6%	47.7%	91.4%	[80,81,82,83]
	SULT1A1*2	G to A change at nucleotide 638	33.2%	29.4%	8%	[80,81,82,83]
	SULT1A1*3	A to G change at nucleotide 667	1.2%	22.9%	0.6%	[80,81,82,83]

**Table 4:** Common genetic variants associated with variation in pharmacokinetics and pharmacodynamics of apixaban.

Apixaban Levels	SNP	Genotype	Locus	Function	Change	Clinical Outcome	Ref.	Year
Peak levels	rs4148738	G>A	*ABCB1*	Intron	Increase	Not tested	[83]	2016
Peak levels and AUC	rs1045642	CC, CT, TT	*ABCB1*	Intron	No significant difference	Not tested	[84]	2018
Peak levels and AUC	rs4148738	CC, CT, TT	*ABCB1*	Intron	No significant difference	Not tested	[84]	2018
Peak levels and AUC	rs776746	CC, CG, GG	*CYP3A5*	Intron	No significant difference	Not tested	[84]	2018

Peak and trough levels of Apixaban and clinical outcomes associated with gene loci and single nucleotide polymorphisms in clinical studies.

**Table 5:** Pharmacogenomic studies of edoxaban.

Clinical Trial	Gene	SNP	DNA Polymorphism	Study Population	Effect on Edoxaban Levels	Clinical Outcomes	Ref.	Year
Integrated analysis of 14 phase I studies	*ABCB1*	rs1045642	C3435T	Healthy population	No effect	Not tested	[93]	2018
Integrated analysis of 14 phase I studies	*SLCO1B1*	rs4149056	T521C	Healthy population	Slight increase in M4 metabolite	Not tested	[93]	2018
Randomized Double blind	*CYP2C9*	rs1799853	---	Venous Thromboembolism	Not tested	No effect	[94]	2017
Randomized Double blind	*CYP2C9*	rs1057910	---	Venous Thromboembolism	Not tested	No effect	[94]	2017
Randomized Double blind	*VKORC1*	rs9923231	---	Venous Thromboembolism	Not tested-	No effect	[94]	2017
Randomized Double blind	*CYP2C9*	rs1799853	---	Atrial Fibrillation	Not tested	No effect	[95]	2015
Randomized Double blind	*CYP2C9*	rs1057910	---	Atrial Fibrillation	Not tested	No effect	[95]	2015
Randomized Double blind	*VKORC1*	rs9923231	---	Atrial Fibrillation	Not tested	No effect	[95]	2015

Pharmacokinetics and clinical outcomes associated with gene loci and single nucleotide polymorphisms tested in clinical studies of Edoxaban.

**Table 6:** Genotype distributions of common variants of *ABCB1* and *CES1.*

Gene	SNP	Genotype	Ethnic Group	*N* %	Minor Allele	MAF%	*p*-Value	Reference
*CES1*	rs2244613	CC	Caucasian	2	C	22.3	0.230	Dimatteo et al. [30]
		CA		37				
		AA		53				
*ABCB1*	rs1045642	CC	Russian	15	T	50.8	0.49	Sychev et al. [52]
		CT		29				
		TT		16				
*ABCB1*	rs4148738	GG	Caucasian	27	A	47.3	0.678	Dimatteo et al. [30]
		AG		43				
		AA		22				
*ABCB1*	rs8192935	CC	Caucasian	43	T	31.5	0.956	Dimatteo et al. [30]
		CT		40				
		TT		9				

MAF: Mean allelic frequency.

## Supplementary Materials

Supplementary materials can be found at https://www.mdpi.com/2075-4426/9/1/7/s1.
