# Supplementary material for: Pharmacogenomics of Novel Direct Oral Anticoagulants: Newly Identified Genes and Genetic Variants
Source: J Pers Med. 2019 Jan 17;9(1):7. doi: 10.3390/jpm9010007 (PMC6463033; doi:10.3390/jpm9010007)
Supplement: Supplementary file 1 [file jpm-09-00007-s001.pdf]

<u>Supplemental Figures</u>

Supplemental Figure 1.

Dabigatran metabolism

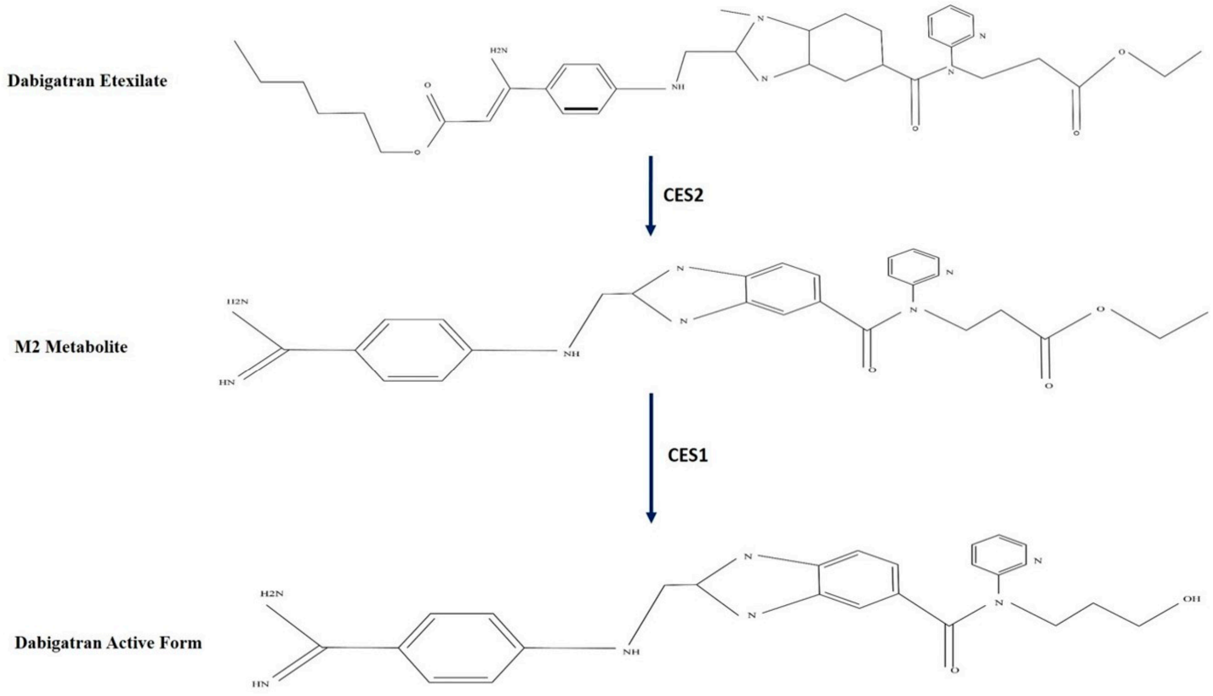

Modified from

Chen, F., et al., *Clinical Implications of Genetic Variation in Carboxylesterase Drug Metabolism*. Vol. 14. 2018. 131-142.

Supplemental Figure 2.

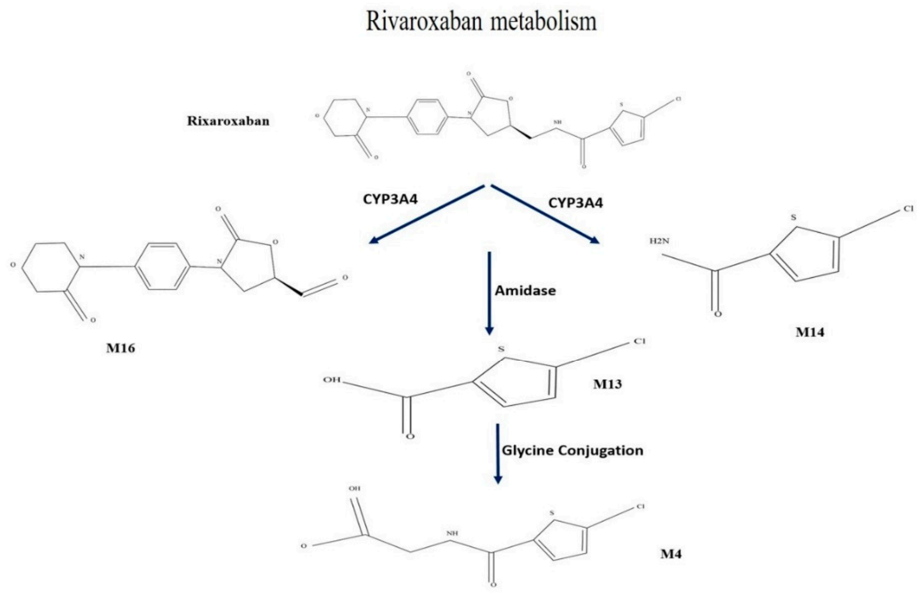

Modified from

Lang, D., C. Freudenberger, and C. Weinz, *In Vitro Metabolism of Rivaroxaban, an Oral, Direct Factor Xa Inhibitor, in Liver Microsomes and Hepatocytes of Rats, Dogs, and Humans.* Drug Metabolism and Disposition, 2009. **37**(5): p. 1046

Supplemental Figure 3.

Apixaban metabolism

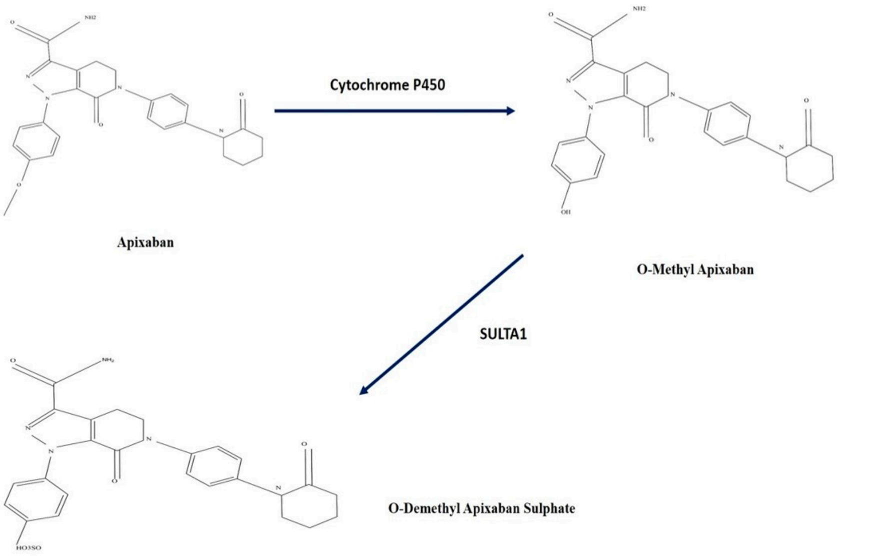

Modified from

Wang, L. et al Sulfation of o-demethyl apixaban: enzyme identification and species comparison. Drug Metabolism Disposition. 2009 Apr;37(4):802-8.

Supplemental Figure 4.

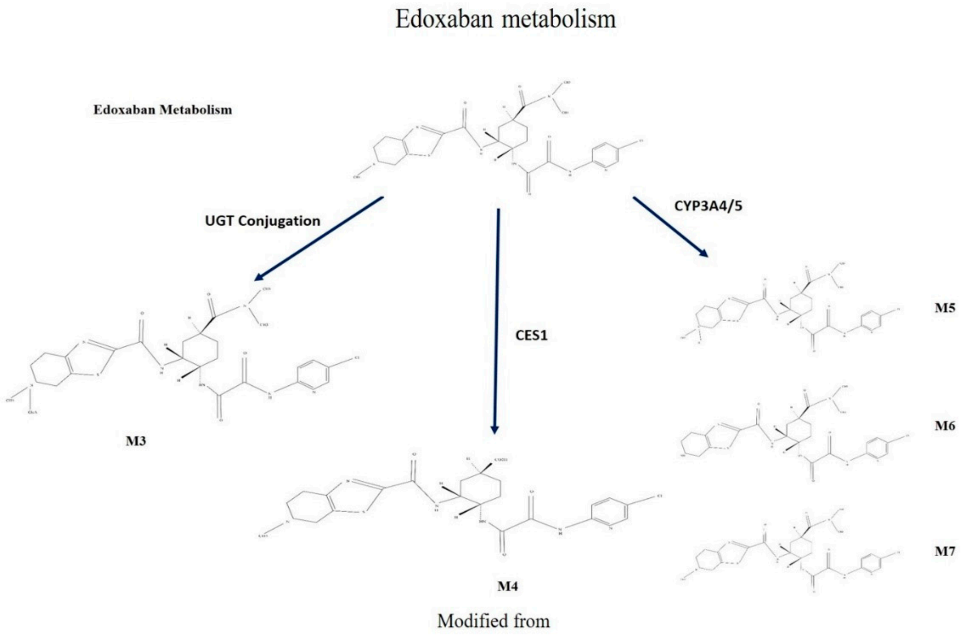

Parasrampuria, D.A. and K.E. Truitt, *Pharmacokinetics and Pharmacodynamics of Edoxaban, a Non-Vitamin K Antagonist Oral Anticoagulant that Inhibits Clotting Factor Xa.* Clin Pharmacokinet, 2016. **55**(6): p. 641-55.
